# Fluid structure interaction of patient specific abdominal aortic aneurysms: a comparison with solid stress models

**DOI:** 10.1186/1475-925X-5-33

**Published:** 2006-05-19

**Authors:** James H Leung, Andrew R Wright, Nick Cheshire, Jeremy Crane, Simon A Thom, Alun D Hughes, Yun Xu

**Affiliations:** 1 Department of Chemical Engineering, Imperial College London, London, UK; 2 Vascular surgery & Radiology, St Mary's Hospital, Imperial College London, London, UK; 3 International Centre for Circulatory Health, National Heart & Lung Institute, Imperial College London, London, UK

## Abstract

**Background:**

Abdominal aortic aneurysm (AAA) is a dilatation of the aortic wall, which can rupture, if left untreated. Previous work has shown that, maximum diameter is not a reliable determinant of AAA rupture. However, it is currently the most widely accepted indicator. Wall stress may be a better indicator and promising patient specific results from structural models using static pressure, have been published. Since flow and pressure inside AAA are non-uniform, the dynamic interaction between the pulsatile flow and wall may influence the predicted wall stress. The purpose of the present study was to compare static and dynamic wall stress analysis of patient specific AAAs.

**Method:**

Patient-specific AAA models were created from CT scans of three patients. Two simulations were performed on each lumen model, fluid structure interaction (FSI) model and static structural (SS) model. The AAA wall was created by dilating the lumen with a uniform 1.5 mm thickness, and was modeled as a non-linear hyperelastic material. Commercial finite element code Adina 8.2 was used for all simulations. The results were compared between the FSI and SS simulations.

**Results:**

Results are presented for the wall stress patterns, wall shear stress patterns, pressure, and velocity fields within the lumen. It is demonstrated that including fluid flow can change local wall stresses slightly. However, as far as the peak wall stress is concerned, this effect is negligible as the difference between SS and FSI models is less than 1%.

**Conclusion:**

The results suggest that fully coupled FSI simulation, which requires considerable computational power to run, adds little to rupture risk prediction. This justifies the use of SS models in previous studies.

## Background

Each year, about 10,000 people in the United Kingdom die of a ruptured abdominal aortic aneurysm (AAA) [1]. AAA is an abnormal dilation of the aorta, and is related to weakening of vessel wall usually as a consequence of atherosclerotic disease. The aorta is the main artery that supplies blood to every vital organ in the body, thus rupture of the aorta can result in catastrophic blood loss leading to death.

Current repair technique is risky [2], hence surgeons adopt a conservative method to operate when the risk of rupture is higher than the risk of surgery. The main clinical indicators used to assess the risk for rupture are the maximum diameter and expansion rate of the AAA, obtained from ultrasound or CT scans. Surgery is recommended when the maximum diameter of AAA measures 55 mm and above or when maximum diameter expands above 10 mm/yr for smaller AAAs [3,4]. Other risk factors, for example, hypertension and smoking, only offer general recommendations and are not usually a justification for surgery [5]. Maximum diameter does have a relationship with the probability of rupture [6], however, the lack of randomized data makes this association unclear [5]. Various clinical studies showed that the risk of rupture of an AAA under 50 mm can range from 12.8%–23% [7]. A screening trial showed that about 5% of the patients in the watchful surveillance group died from aneurysm-related deaths, some after emergency surgery [8]. Clearly, a more accurate indicator is needed in order to reduce the incident of rupture.

Rupture is a mechanical failure when the stress experienced by the vessel wall exceeds wall strength. A patient-specific study have demonstrated that maximum wall stress was 12% more accurate and 13% more sensitive in predicting AAA rupture than maximum diameter [9]. In other patient specific study, peak stress was found significantly higher in ruptured AAA than non-ruptured AAA [10]. In these studies, wall stresses were calculated using solid models with a static uniform internal pressure.

Apart from blood pressure, wall stress in AAA is also influenced by the aneurysm diameter, shape, wall thickness, wall mechanical properties and the presence of thrombus. Studies using idealised fusiform and saccular models showed that wall stress increased with bulge diameter and asymmetry [11]. Moreover, wall stress was found to be more sensitive to wall thickness than asymmetry; an uniform reduction in wall thickness by 25% increased wall stress by ~20% [10]. The effect of thrombus on wall stress has also been investigated [12,13].

However, arterial flow is pulsatile and pressure inside a realistic AAA is non-uniform [14]. The dynamic interaction between flow and wall may influence the predicted wall stress. Di Martino et al. was the first to report patient-specific wall stress results of a fully coupled fluid-solid interaction (FSI) simulation and suggested that the fluid dynamic field could affect wall stress [15]. The choice of whether or not to include fluid motion in AAA stress models depends on what the researcher is looking for in the models. For simulating flow drag force [16], endoleaks [17], and stagnant blood [18] in stented AAAs, the importance of simulating fluid motion is obvious. However, for obtaining peak wall stress as a rupture indicator for surgical management, the views are controversial. The pressure acting on the inner wall is the major determinant of the wall stresses. It is debated that pressure variations, due to fluid motion, can significantly affect wall stress results. Taylor and Yamaguchi have shown in ideal rigid wall models that the vortices at the distal end of the AAA models caused regions of high pressure [19]. However, Finol et al. found in two patient-specific AAA models that hemodynamic pressure variation is insignificant along the inner AAA wall at any stage of the cardiac cycle and that its magnitude and distribution are dependent on the shape and size of an aneurysm [20]. Finol et al., in a later study, compared FSI and structural static simulations on idealised models in order to determine the best suited method to calculate AAA wall stresses [21]. They found that structural models are practical if the peak wall stress is the only subject of interest, since the location of peak stress in the two models were the same. Scottie et al. furthered the study and compared idealised FSI models and static solid models with varying wall thickness and asymmetry [22]. The authors found that static pressure models underestimate wall stress and this effect is most significant in their most asymmetric model. The underestimation was 30.2% for variable wall thickness (0.5 mm-1.5 mm thick), and 10.2% for models with an uniform wall (1.5 mm thick) [22]. Although flow patterns in the asymmetric and axisymmetric models are different, which affect the internal pressure field, their results show that the predicted wall stress is insensitive to flow induced pressure variation.

Papaharilaou et al. used a decoupled FSI approach to study a highly asymmetric 100 mm realistic AAA model with a uniform wall thickness (2.0 mm). For comparison they calculated wall stress by applying a static pressure and found peak wall stress was 12.5% less than the result obtained with the decoupled FSI model [23], which is consistent with Scotties et al.'s finding. The authors further suggested that AAA shape and size have a minor influence on the pressure field compared to the effect of acceleration and deceleration of the flow [23]. Comparing the velocity waveforms between these two studies, the acceleration and initial deceleration of the flow were similar, at ~0.4 m/s^2 ^for acceleration and deceleration. Wolters et al. justified the use of a decoupled approach by arguing that flow induced pressure variation was negligible as it is in the order of 0.1 kPa, compared to the pressure load, which is in order of 10 kPa [24].

It is important to note that Wolters et al [24] and Papaharilaou et al. [23] chose to model AAAs without intraluminal thrombus (ILT). Most large AAAs have ILT [25]; its formation has been linked to platelet exposure to a high and low sequence of wall shear stress (WSS), a common characteristic in AAA [26]. The role of ILT in rupture prevention is controversial. Vorp et al. found that ILT reduced oxygen diffusion to AAA wall, causing local hypoxia and wall weakening [27]. Kazi et al. demonstrated that AAA wall adjacent to ILT was thinner, with smooth muscle cells, and more macrophages and other inflammatory cells than AAA walls without ILT [28]. Nevertheless, studies have shown computationally that ILT reduces peak wall stress [12,13]. Experimental studies suggest that ILT does not reduce pressure on the aneurysm wall [29,30]. Thubrikar et al. found that even though thrombus allows luminal pressure to transfer to the wall, it prevents aneurysm rupture by reducing the strain on the wall [30]. That is, the long-term presence of ILT on rupture is harmful, but the immediate effects are beneficial. It is speculated that the effect of fluid induced pressure variation is likely to be even less when considering a realistic lumen geometry with the presence of ILT.

FSI simulations, compared to models that include the wall only, require more resources and time in terms of computation and data acquisition. Patients would require additional ultrasound or MRI scans for the flow data needed at boundaries. This may cause FSI wall stress analysis to be impractical for large population clinical testing. Hence, it is important to understand the quantitative effect of FSI simulations under current assumptions in order to choose the most efficient model without compromising reliability. The present study was designed to address this issue by comparing results of the FSI and solid models of AAA, constructed from patient-specific data obtained from CT scans.

## Methods

### AAA geometry

Three male patients, all hypertensive and ex-smokers, aged 72, 84, and 77, were selected for this study. All patients have near critical to critical maximum AAA diameters (50 mm, 53 mm, and 57 mm) and modest ILT, with maximum lumen diameters of 32 mm, 42 mm, and 40 mm respectively. The study conformed to the Declaration of Helsinki, and approved by the local research ethics committee. All patients gave written informed consent.

To construct the AAA models for FSI and solid structural simulations, the following information was needed: 1) the geometry of the AAA lumen, 2) the material property of the wall, and 3) the flow conditions at the model boundaries. All patients were given contrast agent and scanned with a spiral CT scanner (Mx 8000 IDT, Philips Electronics, Netherlands), at St Mary's Hospital, London for their routine AAA examinations. Parameters for CT acquisition varied slightly depending on the surgeon's specification or the CT scan operator. For the three patients the parameters were: 240–300 mA, 120 kVp, 4 s scan time for the abdomen, slice spacing 1 – 1.6 mm, 400 mm field of view (FOV), and 512 × 512 image matrix size. This resulted in a resolution of 0.781 mm/pixel. The CT scan was not gated to the cardiac cycle of the patient, hence the reconstruction produced a "time-averaged" AAA geometry.

**Figure 1:**
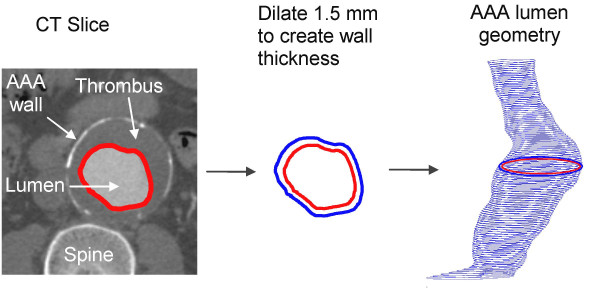
A CT slice: the red line highlights the lumen boundaries found by the region growing algorithm (left). The wall was created by dilating 1.5 mm outward from the boundary of the lumen surface (center). Stacked lumen boundaries create the AAA lumen model (right).

AAA geometries were reconstructed from the entire set of 2D CT slices, starting from the appearance of the renal arteries to the aortic bifurcation, using an in-house Matlab program. The core algorithms of this program was adopted from a previous study [31]. The lumen was the most distinguishable object in a CT image, due to bright contrast agent. The lumen boundaries were segmented automatically by the region growing method (RGM) [32]. This method traces the perimeter of the lumen by seeking pixels of similar intensities (Figure 1, left). Before applying RGM, noise in the image was reduced by using a Gaussian filter, with a 3 × 3 kernel, to clarify the lumen boundaries. The outline of the lumen was fitted by a cubic smoothing spline to remove sharp corners known to create spurious stress levels [33]. Because the lumen borders were obtained automatically, the geometric models reconstructed were reproducible.

FSI simulations are computationally demanding [22]. To simplify the analysis, intraluminal thrombus (ILT) was not included in the models studied here. Instead of having ILT sandwiched between the lumen and wall, an artificial wall was created by dilating the perimeter of the lumen outward by 1.5 mm, the average thickness found in an AAA [15] (Figure 1, center). These models were imported into ADINA 8.2 (Automatic Dynamic Incremental Nonlinear Analysis, Watertown, MA.), using finite element method (FEM) for fluid, solid, and, FSI analysis.

### Boundary conditions and the wall model

**Figure 2:**
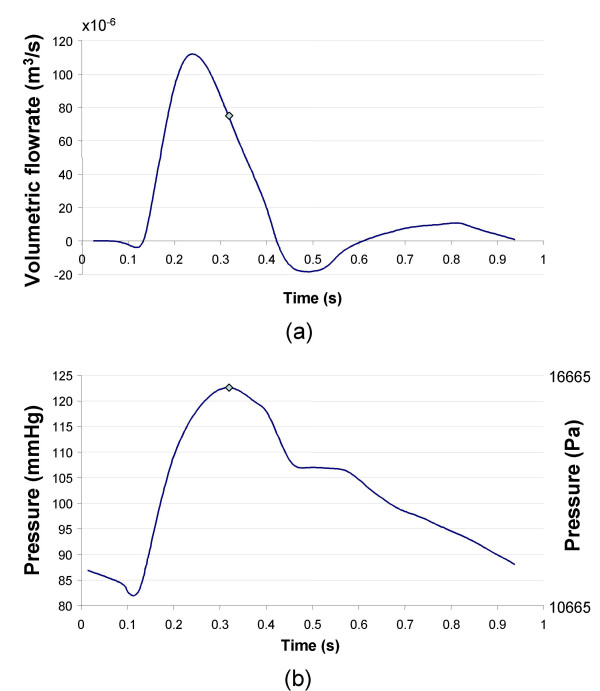
Flow waveform (a), pressure waveform (b) adapted from Olufsen et al. used in FSI [34]. Note 1 cm^3^/s = 0.000 001 m^3^/s and 1 mmHg (0°C) = 133.32239 Pa. Peak flow occurred at 0.24 s, and peak pressure at 0.32 s indicated by the point on each waveform. This was the loading condition for Figures 5,6,8-11.

The Navier-stokes equations and the continuity equations govern the fluid domain. Time dependent flow and pressure waves were based on data acquired by Olufsen et al. from a healthy aorta [34] (Figure 2). Slightly modified versions of both waveforms were used by other patient-specific FSI studies in the literature [16-18,24,35]. The flow and pressure waveforms were applied at the inlet and outlet of the fluid domain respectively. As with most wall stress studies mentioned, the reconstructed AAA geometry was assumed as the zero pressure state. Methods to create a zero pressure state AAA has been proposed to prevent overestimating wall stress [36]. Since overestimation of wall stress would affect all models, the assumed zero pressure model was used for this comparison study. No slip condition was applied at the fluid-solid interface.

The cycle period was 0.94 s, with peak flow occurring at 0.24 s, and peak pressure at 0.32 s. Blood was treated as a homogenous, incompressible, and Newtonian fluid, an acceptable assumption for large arteries [37]. Other properties chosen were dynamic viscosity of 4.00 cP (0.004 Pa s) and density of 1055 kg/m^3 ^(1.055 g/cm^3^) [34].

The flow waveform was applied at the inlet boundary together with a 'plug' flow assumption. During acceleration and early flow deceleration, flow in the infrarenal artery is typically 'plug' [38]. MRI studies found high velocities measured at the infrarenal aorta to skew toward the anterior wall due to the convex curvature of the vessel in that direction [38,39]. However, velocity profiles were observed to be less skewed when measured immediately after the renal arteries, the inlet position of our models [40]. Blood flow was found to be laminar, even during exercise, in asymmetric AAAs [41]; hence a laminar flow assumption was made. The pressure waveform was applied at the outlet as a normal traction. The time-averaged Reynolds number was 730.

The artificial wall bounding the lumen was modeled using the non-linear hyperelastic wall mechanical properties Eq. (1), derived by Ragahvan and Vorp from uniaxial testing of 69 excised human AAAs [42].

*W *= *C*_1_(*I*_*B *_- 3) + *C*_2_(*I*_*B *_- 3)^2 ^    (1)

Where, *W *is the strain energy, and I_B _is the first invariant of the left Cauchy-Green tensor **B **(I_B _= tr **B**). The constants were set to the population mean values C_1 _= 174,000 Pa (17.4 N/cm^2^) and C_2 _= 1,881,000 Pa (1881.1 N/cm^2^). The wall was assumed to be isotropic, with a density of 2000 kg/m^3 ^(2.0 g/cm^3^), Young's modulus of E = 2.7 MPa, Poisson ratio of υ = 0.45, and undergo large displacements. This wall model has been widely used in recent FSI and solid studies [9,10,15,22]. Their results showed that wall stress was relatively insensitive to changes in wall material properties so the mean value was deemed acceptable. To simulate the tethering to the rest of the aorta, both ends of the models were fixed.

FSI simulations were performed using ADINA 8.2 which employs the Arbitrary Lagrangian – Eulerian algorithm (ALE) to couple the solid and fluid domains [43]. For consistency with previous studies, von Mises stress was used for wall stress analysis [9-11,15].

The static pressure models were given an increasing pressure load from 0 to 16341 Pa (122.56 mmHg), the peak pressure of the pressure waveform, over 10 time steps of 0.1 s.

### Numerical discretization

ADINA 8.2 can automatically generate a mesh for any geometry, when proper volumes and subdivisions are prescribed. Each AAA model was divided into quarters intersecting at the center point calculated from each CT slice. This allows free control over the mesh density in any part of the geometry. The subdivisions were optimize, to ensure high quality uniform meshes. Eight-node brick elements were used for the wall. In the fluid domain, eight-node mixed with six-node prisms flow-condition-based-interpolation (FCBI) elements were used to maintain uniform brick shapes. FCBI elements use a linear function to interpolate velocity and a bi-linear function to interpolate pressure and displacement. The solution method for FCBI elements is similar to the finite volume method [43]. The resulting computational mesh had an average element length of 0.7 mm in the solid and 2.0 mm in the fluid domain. The number of elements used depended on the AAA geometry (Table 1).

**Table 1:** Data and results of the study. AAA geometric and mesh details, required computational time and storage, maximum wall stress values and locations from FSI and static pressure simulations. Percentage difference was based on the FSI solution.

	Patient 1	Patient 2	Patient 3
**Max AAA Diameter (mm)**	**57**	**53**	**50**
**Max Lumen Diameter (mm)**	**40**	**42**	**32**
AAA z-axis length (mm)	134	120	96.1
Number of Fluid elements	26,928	25,760	11,628
Number of Solid elements	25,760	13,800	6,800
**Max Wall Stress FSI (Pa)**	**785,126**	**567,969**	**844,014**
**Max Wall Stress Static (Pa)**	**784,052**	**567,508**	**844,908**
**% Difference**	**0.13%**	**0.08%**	**-0.1%**
Location of peak stress, distance from renal arteries (mm)	111	48	72
CPU time static solid simulations (s)	1068	337	762
CPU time FSI (s)	2,135,517	878,210	1,716,848
Hard drive memory storage, gigabytes (GB)	32.5	28.8	14.1

Mesh sensitivity was tested on two AAA geometries by monitoring the magnitude and location of maximum velocity and structural displacement. A mesh density was accepted when the maximum difference in monitored parameters from a denser mesh was less than 5%. This was chosen as a compromise between computational demand and accuracy. Testing two of the three geometries confirmed that the chosen mesh density did not affect the comparison value as both geometries had approximately 5% difference from the mesh independent result.

For the FSI simulation to converge there was a stabilization period, which required increasing pressure from 0–80 mmHg with zero flow for 1 s before applying flow for five cardiac cycles. Time step size was 5 × 10^-3 ^s with 1037 time steps. Newton iteration scheme with 0.001 relative tolerance for degrees of freedom was used. Although the fluid domain required five cardiac cycles, wall stress and displacement reached convergence on the second cardiac cycle. With Adina 8.2, FSI simulations can only be solved using a direct sparse solver. All computations were performed on a 64 bit 1.5 GHz Opteron processor, with 5 GB RAM.

## Results

**Figure 3:**
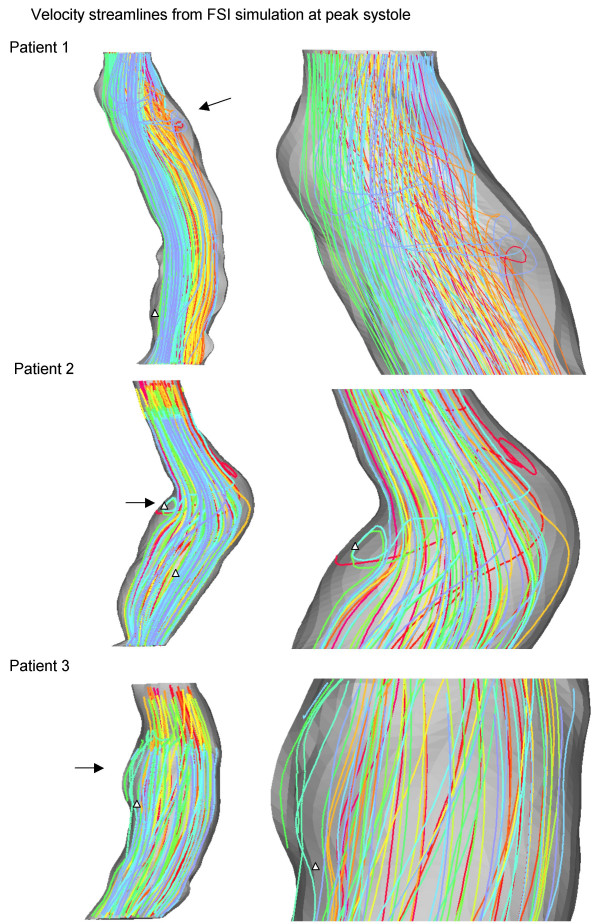
Velocity streamlines at systolic peak (left). Arrows indicate the area of magnification. Magnified image is displayed on the right. Models are not to scale.

**Figure 4:**
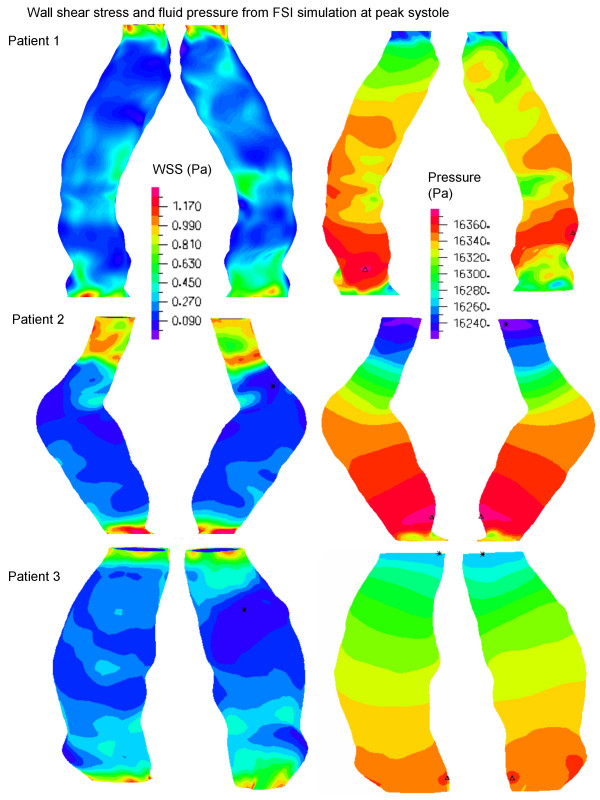
Wall Shear Stress (WSS) distributions at the lumen surface (left) and fluid pressure distribution (right) at systolic peak. Models are not to scale.

Three patients were modeled with the same boundary conditions for FSI simulations to examine the influence of AAA geometry on wall stress. Corresponding static structural models were built to compare with the FSI results. The flow streamlines (Figure 3) and WSS and pressure distributions (Figure 4) are shown at systolic peak for the three FSI models. Artificial high stresses located at the edges were removed. Although the maximum wall diameters were similar among the patients, it can be seen that each AAA has a unique flow pattern, due to the patient-specific shape of the lumen (Figure 3), with vortices and spiraling flow in patients 1 and 2 at peak systole. As shown in Figure 4, WSS was low in the aneurysm bulge due to flow deceleration, and consistent with observations made in both realistic [23,24] and ideal [22] FSI models. At systolic peak, fluid pressure was found to be higher at the distal end, demonstrating the effect of a compliant wall in combination with the physiologic characteristics of the velocity and pressure waveforms. Nevertheless, flow induced pressure variations at peak systole were less than 120 Pa in the three models (Figure 4).

**Figure 5:**
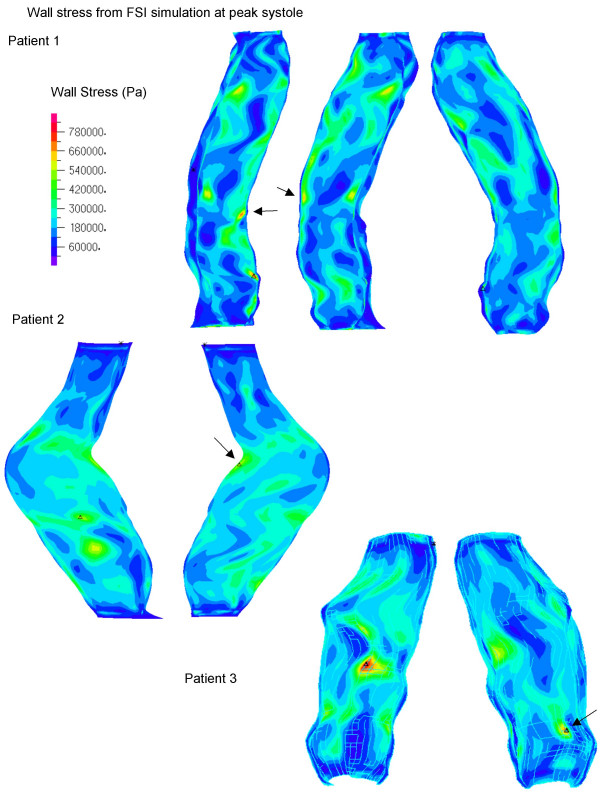
Wall Stress (Pa) patterns of FSI at the Inner surface. The triangles (Δ) indicate peak value of that figure. Arrows indicate secondary high stresses. Note patient 1 has more two points of high stress. Models are not to scale.

**Figure 6:**
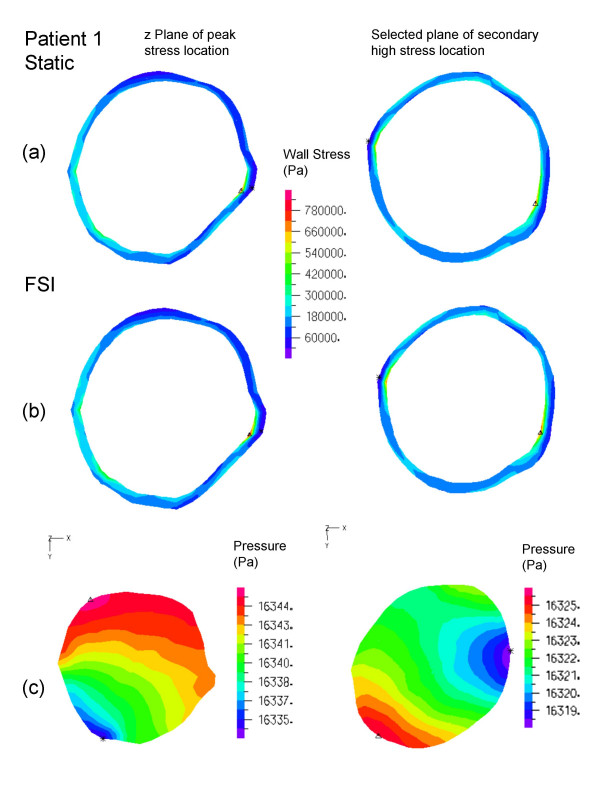
Patient 1, wall stress and fluid pressure in selected planes at the location of peak stress (left) and high stress (right) of the AAA. The inner wall stress of static (top) and FSI (center) are shown with the complementing velocity magnitude (below). The triangles (Δ) indicate maximum stress of that plane.

**Figure 7:**
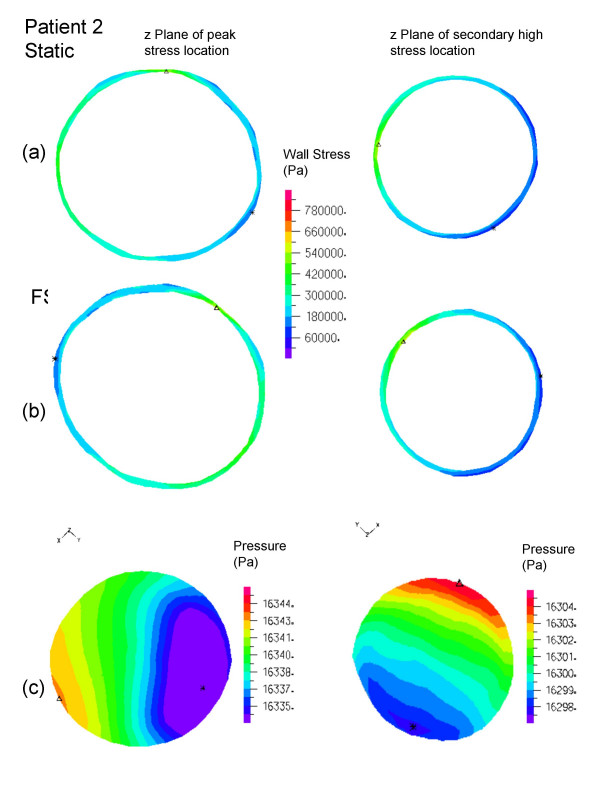
Patient 2, wall stress and fluid pressure in the z-plane at the location of peak stress (left) and high stress (right) of the AAA. The inner wall stress of static (top) and FSI (center) are shown with the complementing velocity magnitude (below). The triangles (Δ) indicate maximum stress of that z plane.

All peak wall stresses occurred at the inner wall, and wall stress patterns were almost identical between the FSI and solid structural models, hence only the FSI stress distributions at peak systole are given here (Figure 5). Isolated high stress spots can be observed in patients 1 and 3 due to irregularity at the lumen surface as a result of ILT. This differs from the peak stress circumferential "belts" reported in a number of patient-specific AAA models [44,45] and ideal models [46] where the surface was much smoother. It could also explain why the stresses were unusually high compared to other FSI studies. The location and value of FSI peak stress and secondary high stresses (Figure 6b, 7b, 8b) were identical to their respective static models (Figure 6a, 7a, 8a). The fluid pressure distributions show little variation across the corresponding cross sectional area (Figure 6c, 7c, 8c). With the FSI model, the location of peak stress remained at the same spot for the majority of the cardiac cycle in patients 1 and 3, suggesting that the wall reacts to pressure instantaneously. Scottie et al. showed a maximum 0.9% difference in peak stress between the pulsatile pressure and static pressure models. This explained why wall stress converged on the second cardiac cycle. With this method of AAA modeling, the wall stress difference between FSI and static models depended only on flow induced pressure variations, which were found to be negligible as compared to the pressure load. Percentage difference in wall stress between the two methods was less than 1% (Table 1).

**Figure 8:**
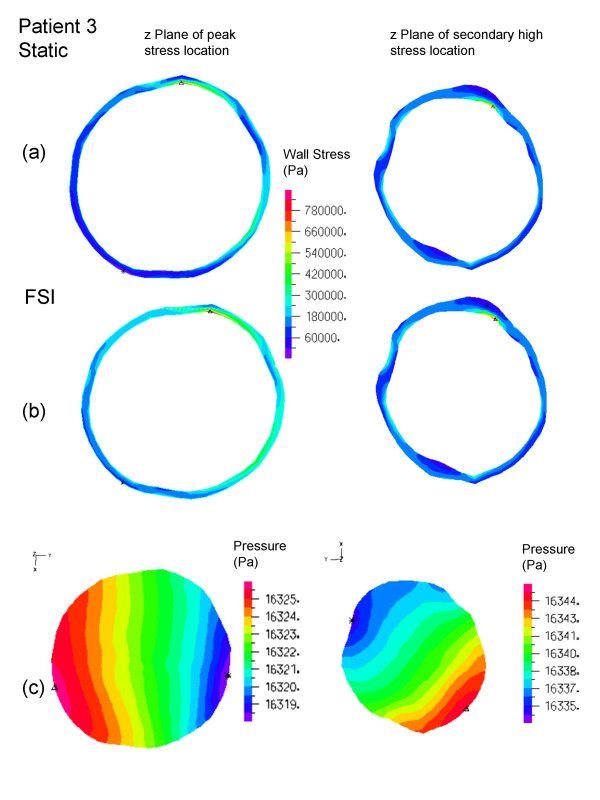
Patient 3, wall stress and fluid pressure in the z-plane at the location of peak stress (left) and high stress (right) of the AAA. The inner wall stress of static (top) and FSI (center) are shown with the complementing velocity magnitude (below). The triangles (Δ) indicate maximum stress of that z plane.

### Limitation

The stress values presented here do not represent the actual stress experienced in the wall, since the model used an assumed zero pressure state, and ILT and calcification were not included. The percentage difference in peak wall stress between the FSI and solid structural models was lower than previous comparative studies [22,23], due to the higher peak wall stress levels resulted from surface irregularity. Doppler ultrasound velocity measurements were made on these patients and using patient-specific velocity waveforms resulted in a difference of about 3% from the static pressure models.

**Figure 9:**
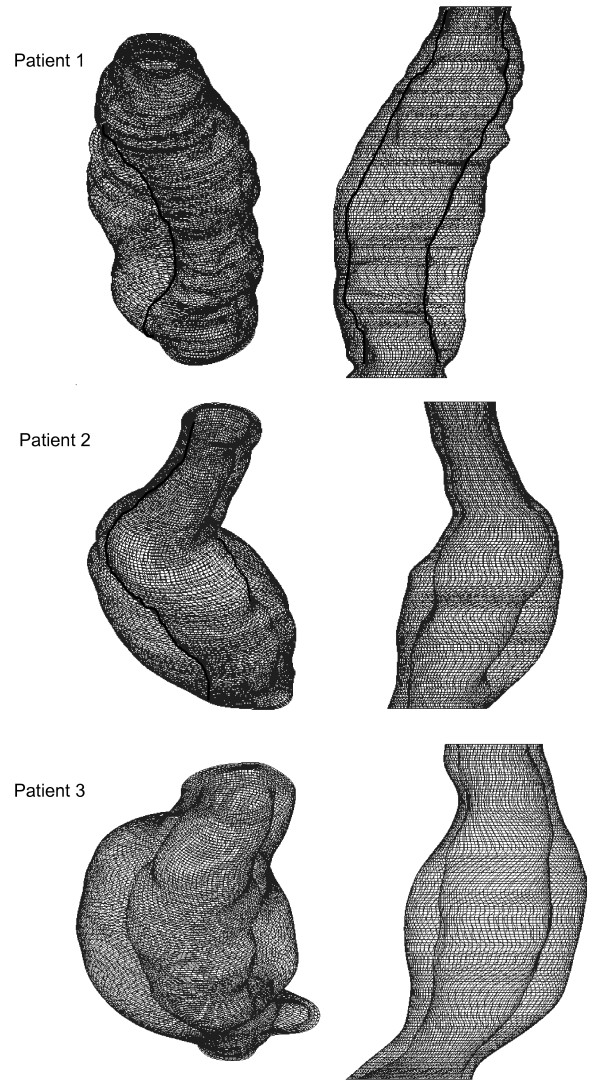
Transparent geometries of the entire aneurysm showing thickness of the wall and thrombus surrounding the lumen of each patient.

Another important limitation of the present study is the assumed uniform wall thickness. The patients studied here have very thick ILT (Figure 9), the 'wall' should have included ILT as well as the arterial wall. It has been shown that ILT can reduce the strain and rate of dilation by up to 15% [30]. Furthermore, variation in arterial wall thickness have a greater influence than variations in the wall material models [10,47]. However, it was found that the average wall thickness of AAAs were 1.5188 mm (for maximum AAA diameters <70 mm) and 1.402514 mm (for maximum AAA diameters >80 mm) with an absolute minimum wall thickness of 1.40 mm in both groups[48]. Bixaxial wall models is available [49] and it is predicted that this model will increase circumferential wall stresses than Raghavan and Vorp's model [42]. On the other hand, Williamson et al. found stresses within the arterial wall were insensitive to variations in the elastic modulus, and to other wall features, such as fibrous plaque, calcified plaque, and lipid pools [50].

Wall strength plays the other role of restraining rupture. It is possible for a location with lower than peak stress to rupture due to lower wall strength [51]. Vorp and Vande Geest provided preliminary results on estimating the combination of wall strength and stress [7].

Finally, the relationships between wall stress, wall strength, and the behavior of living tissue requires further work. Sakalihasan et al. used a PET scan to determine metabolic activity in the AAA wall and found that high metabolic activity, measured by sugar uptake, can predict rupture with 90% accuracy [52]. We aim to determine the relationship between high wall stress and metabolic activity in future studies.

## Conclusion

This was a comparative investigation of FSI and solid modeling of three AAA patients. It has been shown that flow induced pressure variations were too small to cause a noticeable difference in wall stress. Since the time required for an FSI simulation is 3 to 4 orders of magnitude greater than the solid structural simulation, we suggest that solid model with a static pressure corresponding to the peak systolic pressure would be sufficient for wall stress prediction.

## Declaration of competing interests

The author(s) declare that they have no competing interests.

## Authors' contributions

JL coded the image segmentation program, created AAA models, conducted the simulations, analyzed the results, and prepared the manuscript. XX designed and supervised the study, and revised and gave final approval of the manuscript. ST designed and coordinated the clinical part of the study. AH participated in the design of the study and revised the manuscript. JC coordinated the retrieval of the patient's consent and medical reports, and approved the ultrasound scanning. NC participated in the design of the study and approved the selection of his patients. AW acquired and interpreted the CT scans of the patients. All authors read and approved of the manuscript.
